# T2 mapping for the characterization of prostate lesions

**DOI:** 10.1007/s00345-022-03991-8

**Published:** 2022-03-31

**Authors:** Tobias Hepp, Laura Kalmbach, Manuel Kolb, Petros Martirosian, Tom Hilbert, Wolfgang M. Thaiss, Mike Notohamiprodjo, Jens Bedke, Konstantin Nikolaou, Arnulf Stenzl, Stephan Kruck, Sascha Kaufmann

**Affiliations:** 1 Department of Diagnostic and Interventional Radiology, University Hospital Tübingen, Hoppe-Seyler-Str. 3, 72076 Tübingen, Germany; 2 Department of Urology, University Hospital Tuebingen, Hoppe-Seyler-Str. 3, 72076 Tübingen, Germany; 3 Clinic of Urology, Siloah St. Trudpert Klinikum, Wilferdinger Str. 67, 75179 Pforzheim, Germany; 4 Empirical Inference Department, Max Planck Institute for Intelligent Systems, Max-Planck-Ring 4, 72076 Tübingen, Germany; 5 Diagnostic and Interventional Radiology, Siloah St. Trudpert Klinikum, Wilferdinger Str. 67, 75179 Pforzheim, Germany; 6 Advanced Clinical Imaging Technology, Siemens Healthcare AG, Lausanne, Switzerland; 7 Department of Radiology, Lausanne University Hospital and University of Lausanne, Lausanne, Switzerland; 8 LTS5, Ecole Polytechnique Fédérale de Lausanne (EPFL), Lausanne, Switzerland; 9 Department of Nuclear Medicine, University Hospital Ulm, Albert-Einstein-Allee 23, 89081 Ulm, Germany

**Keywords:** Prostate cancer, Prostatitis, Magnetic resonance imaging, T2 mapping, Image-guided biopsy

## Abstract

**Purpose:**

Purpose of this study is to evaluate the diagnostic accuracy of quantitative T2/ADC values in differentiating between PCa and lesions showing non-specific inflammatory infiltrates and atrophy, features of chronic prostatitis, as the most common histologically proven differential diagnosis.

**Methods:**

In this retrospective, single-center cohort study, we analyzed 55 patients suspected of PCa, who underwent mpMRI (3T) including quantitative T2 maps before robot-assisted mpMRI-TRUS fusion prostate biopsy. All prostate lesions were scored according to PI-RADS v2.1. Regions of interest (ROIs) were annotated in focal lesions and normal prostate tissue. Quantitative mpMRI values from T2 mapping and ADC were compared using two-tailed *t* tests. Receiver operating characteristic curves (ROCs) and cutoff were calculated to differentiate between PCa and chronic prostatitis.

**Results:**

Focal lesions showed significantly lower ADC and T2 mapping values than normal prostate tissue (*p* < 0.001). PCa showed significantly lower ADC and T2 values than chronic prostatitis (*p* < 0.001). ROC analysis revealed areas under the receiver operating characteristic curves (AUCs) of 0.85 (95% CI 0.74–0.97) for quantitative ADC values and 0.84 (95% CI 0.73–0.96) for T2 mapping. A significant correlation between ADC and T2 values was observed (*r* = 0.70; *p* < 0.001).

**Conclusion:**

T2 mapping showed high diagnostic accuracy for differentiating between PCa and chronic prostatitis, comparable to the performance of ADC values.

**Supplementary Information:**

The online version contains supplementary material available at 10.1007/s00345-022-03991-8.

## Introduction

Prostate cancer (PCa) is the most common non-cutaneous cancer among men with a lifetime risk of up to 37% [1]. Multiparametric magnetic resonance imaging (mpMRI) is presently regarded as the best imaging modality for detection and localization of prostate cancer (PCa) [2]. Further standardization due to the introduction of the Prostate Imaging–Reporting and Data System (PI-RADS) based on multiple tissue characteristics increased the cancer detection rate [3]. Currently, PI-RADS v2.1 conform mpMRI includes T2-weighted sequences (T2w), diffusion-weighted imaging (DWI) and dynamic contrast-enhanced imaging (DCE) [2]. In targeted prostate biopsy of a lesion with malignant imaging findings one of the most common benign pathologic findings beside PCa is chronic prostatitis. Especially in case of PI-RADS 3 lesions, histopathology shows about 50% chronic prostatitis [4, 5]. However, differentiating PCa from chronic prostatitis in mpMRI can be challenging. Areas affected by chronic prostatitis in the peripheral zone (PZ) can be associated with reduced signal intensity in T2w images and apparent diffusion coefficient (ADC) maps, which are derived from DWI. The classical shape of chronic prostatitis in MRI is sometimes replaced by focal or irregular appearances, mimicking PCa. Furthermore, PCa and chronic prostatitis are both often accompanied by elevation of serum prostate-specific antigen (PSA) [5–7].

T2w fast spin-echo (FSE) sequences provide images with high spatial resolution of the prostate that allow the depiction of its zonal anatomy [8]. However, signal intensities may vary locally as a result of radio-frequency inhomogeneities of the receiver coil and are thus not suitable as a quantitative parameter. By contrast, quantitative T2 values generated by T2 mapping are independent of such signal variations and reflect the absolute values of the T2 relaxation time. Therefore, including T2 relaxation time as an absolute quantitative parameter for further characterization of focal prostate lesions sounds promising. Several studies have already evaluated quantitative parameters including T2 values, but most of them included only patients with histology-proven prostate cancer and varying methods of histopathological reference standards [9–12]. Regarding this, the question arises whether T2 values can be used to differentiate between PCa and chronic prostatitis in a typical clinical cohort of men. In this study, T2 values for PCa, chronic prostatitis and normal tissue were analyzed and the diagnostic accuracy of T2 values was compared with ADC values. Histopathology derived from robot-assisted mpMRI-TRUS fusion prostate biopsy (RA-TB) was used as reference.

## Materials and methods

This retrospective, single-center cohort study was approved by the institutional review board (359/2019BO2). Between January 2015 and March 2018, we enrolled a total of 63 patients meeting the following inclusion criteria (a) suspected for PCa; (b) mpMRI (T2w imaging, DWI and DCE imaging including T2 mapping at 3T); (c) PI-RADS v2.1 Score ≥ 3; (d) histopathological reference obtained by RA-TB and (e) available PSA value measured previously to the biopsy. Exclusion criteria were (a) relevant motion artifacts on MRI images (*n* = 4), (b) technical problems with postprocessing and export of MRI datasets (*n* = 2) and (c) two patients with elevated PSA levels (51 and 63 ng/ml, respectively) were excluded due to a high risk of prostate cancer, which was subsequently also confirmed by biopsy. Eventually, we included 55 patients in our analysis.

### MR imaging

All patients underwent mpMRI according to the European Society of Urogenital Radiology guidelines. PI-RADS v2.1 classification was used to describe the lesions found. All mpMRI examinations were performed at the same center using a 3T scanner (MAGNETOM Skyra, Siemens Healthcare, Erlangen, Germany).

Standard T2w imaging was performed using fast spin-echo sequences. EPI-sequences were used for DWI. Additional to these standard sequences we routinely add a vendor provided prototype model-based accelerated T2 mapping sequence that uses voxel-wise measurements of tissue T2 relaxation time values [13]. Detailed MR acquisition parameters are reported in Table S1 of the Supplementary Information.

### Robot-assisted mpMRI-TRUS fusion prostate biopsy [14]

All included patients underwent prostate biopsy with the iSR’obot Mona Lisa robot unit using the UroBiopsy™ 3D modelling software (both: Biobot Surgical, Singapore) with an ultrasound machine (Pro Focus 2202, BK Medical, Peabody, MA, USA) and a multi-frequency ultrasound probe (BK 8848, BK Medical, Peabody, MA, USA) in lithotomy position [14]. First, targeted biopsy samples were obtained for the suspect lesion known from mpMRI. These targeted lesions were identified in consensus by two board certified uroradiologists with 5 and 12 years of experience (WT and SK). Second, we obtained an off-target computer-guided transperineal template saturation biopsy of multiple tissue samples without covering the mpMRI target lesion, while emphasizing the PZ. All procedures were performed by an experienced urologist consultant. All gathered tissue samples were fixated using formalin solution and evaluated by two experienced uro-pathologists.

### Quantitative T2 values correlated with histopathology

To determine the quantitative T2 and ADC values from the lesions that were sampled through targeted biopsy, we defined regions of interest (ROI) in the suspicious solitary, circumscribed lesions previously identified for and histologically proven by the RA-TB. The ROI was annotated voxel-wise for a single slice with maximum extent of the suspicious lesion.

Reference ROIs were annotated in the PZ of the prostate on the same slice as the lesion, if it was not suspicious (PI-RADS 1), or in the PZ on a neighboring slice in case of signal alterations (≥ PI-RADS 2).

For all ROIs, the mean signal intensity of the ADC and T2 maps were calculated over all included voxels. NORA (Nora Medical Imaging Platform Project, University Medical Center Freiburg, Germany) was used to read the images and to annotate all ROIs.

### Statistical analysis

Statistical analysis was performed using SPSS 26 (IBM Corp. Released 2019. IBM SPSS Statistics for Windows, Version 26.0. Armonk, NY, USA). Characteristics and quantitative mpMRI values of subpopulations were compared using two-tailed t-tests. The results are given as mean ± standard deviation. ROC analysis was performed and Youden’s index was used to select the optimum cutoff point. The area under the receiver operating characteristic curve (AUC) and its 95% confidence interval (CI) were calculated. Correlation between mean T2 and mean ADC values was evaluated using Pearson’s correlation. Statistical significance was considered at *p* < 0.05.

## Results

### Patient characteristics

We included a total of 55 patients in our study (mean age 63.8 ± 7.4 years). PCa was detected in 29 patients, while the histopathological examination of the other 26 lesions showed non-specific inflammatory infiltrates and atrophy, features of chronic prostatitis (18 of 29 patients with PCa and 14 of 26 patients with prostatitis were biopsy-naive). There was no significant age difference between the two groups (*p* = 0.08).

The mean PSA level of all patients combined was 8.6 ± 3.4 ng/ml. PSA was slightly higher in patients with PCa (9.3 ± 3.3 ng/ml vs. 7.7 ± 3.4 ng/ml). However, this difference was not statistically significant (*p* = 0.07).

**Fig. 1:**
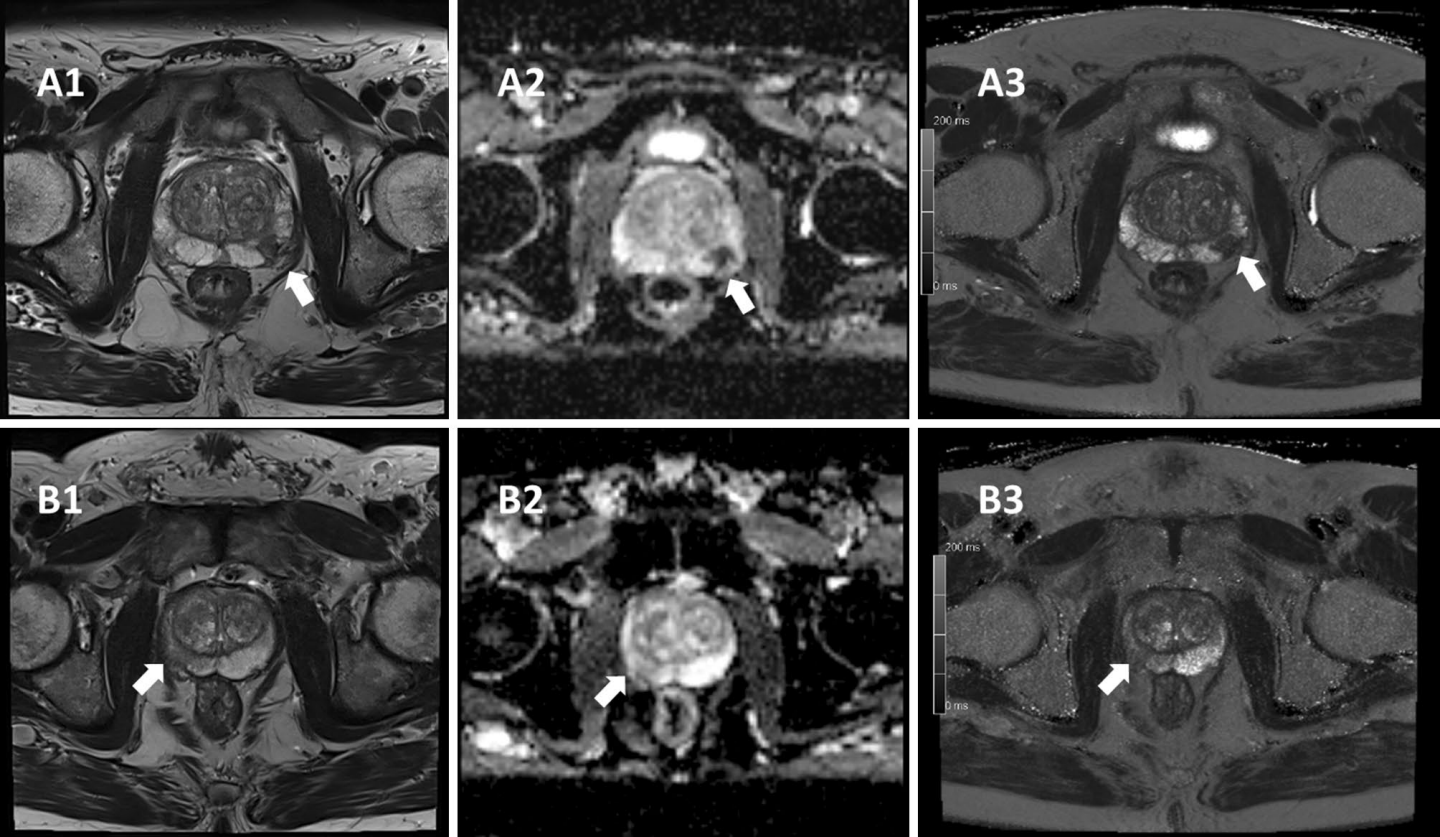
Patient A was referred with a PSA of 10.0 ng/ml. He was scored PI-RADS 4 in the left PZpl mid region according to PI-RADS v2.1. Histopathology revealed a high-grade prostate carcinoma with the highest Gleason score of 8. Patient A underwent radical prostatectomy, and final histopathology confirmed the results of the biopsy. The patient was staged pT2c pN0 cM0 according to the current TNM classification. Picture A1 shows regular T2-weighted MRI with a circumscribed hypointense lesion in the left peripheral zone of the mid prostate without penetrating growth. Picture A2 shows standard ADC map with a value of 0.43 × 10^–3^ mm^2^/s. The reference value for normal tissue was 1.94 × 10^–3^ mm^2^/s. Picture A3 shows T2 mapping of the same lesion with a value of 61 ms. The reference value for normal tissue was 284 ms. Patient B was referred with a PSA of 4.8 ng/ml. He was scored PI-RADS 4 in the right PZpl mid region according to PI-RADS v2.1. Histopathology revealed chronic prostatitis. Patient B did not undergo oncological therapy. Picture B1 shows regular T2-weighted MRI with a circumscribed hypointense lesion in the right peripheral zone of the mid prostate without penetrating growth. Picture B2 shows standard ADC map with a value of 1.12 × 10^–3^ mm^2^/s. The reference value for normal tissue was 1.89 × 10^–3^ mm^2^/s. Picture B3 shows T2 mapping of the same lesion with a value of 132 ms. The reference value for normal tissue was 311 ms

The average delay between MRI examination and RA-TB was 31 ± 34 days. Among the 29 cases of PCa, two exhibited a Gleason of 6, nine a Gleason of 7a, six a Gleason of 7b, and twelve a Gleason of 8 or above. An example of two patients with PCa, respectively chronic prostatitis is given in Fig. 1.

### mpMRI results

According to the evaluation of standard mpMRIs using PI-RADS v2.1, the most frequent PI-RADS score in patients with PCa was 4 and in patients with chronic prostatitis 3, respectively (Table 1).

**Table 1:** Distribution of PI-RADS scores

PI-RADS score	All	Prostate cancer	Chronic prostatitis
3	18	7 (39%)	11 (61%)
4	23	13 (57%)	10 (43%)
5	14	9 (64%)	5 (36%)

**Fig. 2:**
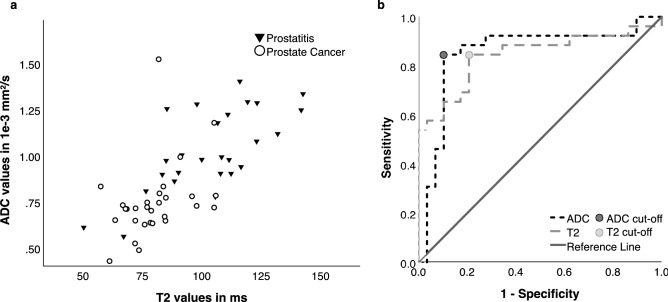
**a** ADC and T2 values for all pathological lesions in conjunction with the decision boundary derived from multivariable binary logistic regression (ADC values in 10^–3^ mm^2^/s; T2 values in milliseconds). **b** Discrimination between prostate cancer and chronic prostatitis. Receiver operating characteristic curves for ADC values and T2 values

Pathological lesions (PCa or chronic prostatitis) in general had significantly lower T2 values than control parenchyma (91 ± 21 ms vs. 193 ± 58 ms; *p* < 0.001). Within those pathological lesions PCa showed significantly lower values than chronic prostatitis (80 ± 13 ms vs. 104 ± 22 ms; *p* < 0.001). ROC analysis revealed an AUC of 0.84; CI [0.73–0.96] and a T2 threshold of 84.98 ms (sensitivity 84.6%/specificity 79.3%) for the classification of PCa and chronic prostatitis (Fig. 2).

In quantitative ADC analysis, pathological lesions (PCa and chronic prostatitis) had a significantly lower value than the control parenchyma (0.88 ± 0.25 × 10^–3^ mm^2^/s vs.1.59 ± 0.23 × 10^–3^ mm^2^/s; *p* < 0.001). Within those pathological lesions PCa showed significantly lower values than chronic prostatitis (0.75 ± 0.20 × 10^–3^ mm^2^/s vs. 1.03 ± 0.22 × 10^–3^ mm^2^/s; *p* < 0.001). ROC analysis revealed an AUC of 0.85; CI [0.74–0.97] and a cutoff value of 0.85 × 10^–3^ mm^2^/s (sensitivity 84.6% / specificity 89.7%) to differentiate between PCa and chronic prostatitis (Fig. 2).

T2 and ADC values of pathological proven lesions showed a significant positive correlation of 0.70 (*p* < 0.001) (Fig. 2).

## Discussion

One of the challenges of mpMRI is to differentiate between so-called chronic prostatitis and PCa in patients with elevated PSA. PI-RADS scoring enables a precise description of prostate lesions. However, chronic prostatitis represents a common source of false-positive findings in mpMRI. The differentiation between PCa and chronic prostatitis is particularly complicated for lesions scored as PI-RADS 3 or 4, and both entities are the most common histopathological correlates of these kind of lesions [5, 15].

Our study has shown that the discrimination between normal and pathological tissue as well as PCa and chronic prostatitis is feasible using both T2 and ADC values.

The quantitative values for PCa were significantly lower than those for chronic prostatitis. Similar AUCs of T2 and ADC with highly overlapping CIs demonstrate a comparable diagnostic accuracy of both parameters. There is no sufficient evidence that ADC outperforms T2 in differentiating PCa and chronic prostatitis.

The T2 values measured in this study are consistent with previously reported values for PCa and normal prostate tissue [11, 16–20].

Mai et al. [12] evaluated T2 mapping of the prostate using the same technique. However, no actual T2 values were reported for cases with chronic prostatitis. In a subgroup, the distinction between PCa and chronic prostatitis was evaluated performing ROC analysis with an AUC of 0.85 for T2 values and 0.84 for ADC maps, respectively. These values show high correspondence with our results. To the best of our knowledge, this study is the only one that has so far investigated the differentiability of PCa and chronic prostatitis using the same technique to acquire quantitative T2 values.

Even though the establishment of PI-RADS led to an improved standardization of prostate MRI interpretation, inter-reader variability is still a limitation of the system [21]. By means of quantitative parameters, objectivity in image analysis could be increased. Furthermore, quantitative values are essential for the development and application of computer-aided detection systems supporting radiological diagnostics [22, 23].

Until now, T2 mapping was mostly restricted to research purposes. The underlying reason for this constricted application might be the prolonged acquisition time of T2 maps as well as the more complex postprocessing compared to standard T2w imaging [8, 18, 22]. Different efforts were undertaken to shorten scan times for T2 mapping [17, 18, 22]. This could facilitate their integration into clinical routine imaging.

In addition, Mai et al. indicated that calculated T2w images derived from model-based accelerated T2 mapping are equivalent to the conventional T2w images in terms of anatomical and diagnostic accuracy [12]. Considering these findings, it can be summarized that T2 mapping is able to provide the detailed morphological information of a T2w sequence via calculated images. Therefore, one might hypothesize, that T2 mapping with calculated T2w images could be able to replace standard T2w imaging in mpMRI. On the other hand, T2 mapping additionally provides a quantitative biomarker, which characterizes the biology of prostate tissue. The predictive value of T2 mapping in different diagnostic settings has been shown in the last years: For instance, Roux et al. and Jan Fritz used T2 mapping for musculoskeletal assessment of the knee [24]. Li et al. used T2 mapping in an oncological setting for cervical cancer [25].

Because of the significant correlation of T2 and ADC values, which has also been confirmed by other studies [12, 26, 27], we were not able to yield a distinct information gain by combining both quantitative parameters. It has been indicated that the underlying pathophysiological background of this correlation might be the shared dependency of T2 and ADC values on cell density [26, 28]. This is also illustrated by the result of our ROC analysis, which demonstrates a comparable diagnostic accuracy for the discrimination between PCa and chronic prostatitis for T2 and ADC values.

However, our results cannot yet be considered sufficient to conclude that T2 mapping can be regarded as equivalent to ADC values, which are used routinely in PCa diagnostics, nor if T2 mapping will possibly replace this established technique.

A limitation to keep in mind is that we used T2w images and DWI/ADC to identify prostate lesions, which introduces a certain bias. Especially within the PZ, the sensitivity of detecting PCa is significantly higher for T2w MRI combined with DWI/ADC than for T2w imaging alone [29]. Therefore, it could well be possible to miss pathological lesions if DWI, including the derived ADC map, is omitted.

This study must be interpreted within its limitations. The retrospective nature of this study might precipitate potential bias. The number of examined patients is rather small, but we were aiming for a high homogeneity of our study population to present reliable results. It must be taken into consideration that the ROIs were annotated manually which could be a source of error. Although the chosen biopsy technique shows high reliability [14], sampling errors might still have occurred.

In conclusion, we can summarize that T2 mapping and ADC values are both valuable and show similar diagnostic accuracy for differentiating between PCa and chronic prostatitis. Therefore, T2 mapping with calculated T2w images could replace standard T2w imaging in mpMRI and additionally provide a quantitative biomarker characterizing prostate tissue properties.

## Supplementary Information

Below is the link to the electronic supplementary material.

Supplementary file1 (XLSX 11 KB)

## Data Availability

Data are not publicly available.

## Code availability

Code is not publicly available.
